# Electrochemical and Computational Studies Show That Vitamin C Assists Resveratrol, Piceatannol and Oxyresveratrol in Superoxide Scavenging, Suggesting a Superoxide Dismutase Mechanism

**DOI:** 10.3390/ijms27135691

**Published:** 2026-06-24

**Authors:** Francesco Caruso, Taylor S. Teitsworth, Raiyan Sakib, Alessio Caruso, Stuart Belli, Miriam Rossi

**Affiliations:** Vassar College, Chemistry Department, Poughkeepsie, NY 12604, USA; tteitsworth@vassar.edu (T.S.T.);

**Keywords:** resveratrol, piceatannol, oxyresveratrol, superoxide, antioxidant, RRDE, DFT

## Abstract

In this study, we combine experimental and computational approaches to elucidate a density functional theory (DFT)-derived mechanism for superoxide scavenging by resveratrol, piceatannol, and oxyresveratrol. Using rotating ring–disk electrode (RRDE) hydrodynamic voltammetry, the superoxide radicals are generated *in situ*, allowing direct measurement of antioxidant activity. Data show that the catechol-containing piceatannol is approximately four times more active than resveratrol, while resveratrol and oxyresveratrol exhibit similar efficiencies, indicating that the additional 2′-OH group in oxyresveratrol has minimal impact. Vitamin C (ascorbic acid) facilitates scavenging by acting as a proton donor for resveratrol, piceatannol, and 4′-OH oxyresveratrol, but it is unable to deprotonate the 2′OH group of oxyresveratrol. The experimental results suggest a superoxide dismutase (SOD)-like mechanism, obtained from energetically feasible DFT calculations, in which these stilbenes convert two superoxide anions into H_2_O_2_ and O_2_, helped by vitamin C. Mechanistically, the first superoxide is reduced by abstracting a hydroxyl-group hydrogen atom, while the second undergoes oxidation via π–π interaction with the aromatic system, releasing O_2_. Notably, resveratrol can be regenerated through a catalytic cycle involving vitamin C. These data underscore the SOD-mimicking properties of dietary polyphenols and suggest a need to reevaluate resveratrol’s clinical utility regardless of its low bioavailability.

## 1. Introduction

It is widely accepted that dysfunction in cellular concentration of reactive oxygen species (ROS) is implicated in many diseases and in aging characteristics. Endogenous superoxide (O_2_^●−^) is a precursor to other ROS and primarily produced by one-electron reduction of molecular oxygen obtained from the mitochondrial electron transport chain [1]. When these reactive species overwhelm the body’s natural detoxification systems, it results in oxidative stress. The superoxide dismutase family of metalloenzymes serves as the body’s primary antioxidant defense against superoxide damage under these conditions. Of these, manganese superoxide dismutase (MnSOD) is the only SOD isoform existing in the mitochondrial matrix that can decrease O_2_^●−^ concentration to avoid cellular damage [2]. The mechanistic details for how the Mn metal ion deals with O_2_^●−^—by oxidation to O_2_ (with Mn^3+^ ion) and by reduction to H_2_O_2_ (with Mn^2+^ ion)—were recently explained by the Borgstahl group [3]. Boosting the body’s enzymatic defense systems through a healthy diet is considered a good strategy to contend with oxidative stress. Recently, an extensive study [4] linked specific dietary patterns to healthy aging, highlighting high consumption of fruits and vegetables alongside moderate wine intake. These patterns correlate with the presence of small dietary molecules capable of eliminating ROS [5].

**Figure 1:**
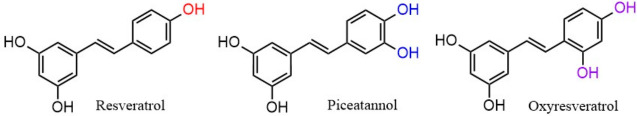
The three naturally occurring phenolic stilbenes, resveratrol, piceatannol and oxyresveratrol. Reactive hydroxyls indicated in color.

Stilbenes, such as resveratrol, piceatannol and oxyresveratrol (Figure 1), are among the natural compounds that are prevalent in fruits including peanuts, mulberries, blueberries, raspberries, grape skins and wines [6]. These compounds are secondary metabolites produced by certain plants such as *Vitis vinifera* and *Polygonum cuspidatum* (Japanese knotweed) under conditions of oxidative stress, such as exposure to pathogens or ultraviolet light [7]. *Polygonum cuspidatum*, which has a notably high concentration of resveratrol, is used in traditional Chinese medicine [8] to treat several ailments. As seen with many plant-derived natural products, these stilbene derivatives demonstrate important beneficial biomedical effects on human health [9]. For example, resveratrol is a strong antioxidant [10] that shows anti-aging [11,12,13], anti-inflammatory features [14] and displays potential neuroprotective effects in experimental and animal models of Alzheimer’s and Parkinson’s diseases [15,16,17,18]. Polyphenolic compounds, specifically stilbene derivatives, are effective neuroprotective agents against cerebral ischemia–stroke [19] and because resveratrol can permeate the blood–brain barrier, it can directly lessen ischemic damage [20]. Resveratrol and its derivative, oxyresveratrol, also show activity against some skin disorders such as skin cancer, photoaging, dermatitis, and melanogenesis [21,22,23]. In vitro studies on human skin epidermal cells demonstrated that combining resveratrol and vitamin C helps protect and maintain healthy skin [24]. Resveratrol action on cardiovascular diseases (CVDs) has been recently reviewed [25,26].

The beneficial role of resveratrol towards CVD received prior attention due to its association with the heart-protective French paradox [27,28]. In fact, a recent study associates lower CVD in persons following the Mediterranean diet with moderate wine consumption by measuring microbial phenolic metabolites to objectively quantitate wine use [29,30]. Additionally, resveratrol and its analogs are strong activators of the sirtuin family of proteins and imitate caloric restriction [31,32], thereby fostering anti-aging effects.

Several clinical trials to study the therapeutic potential of resveratrol (which is non-toxic), using dosages of up to 1 g/day, mostly focused on pathologies related to oxidative stress such as type 2 diabetes, cardiovascular diseases, and neurological decline [33,34]. The results of these trials demonstrated promising benefits due to improved antioxidant activity by resveratrol and its ability to modulate neuroinflammatory effects. Other clinical trials show its ability to reduce blood pressure to normal levels without additional antihypertensive drugs and possible prevention of liver damage [35,36]. Consistent with other resveratrol derivatives, oxyresveratrol exhibits antioxidant and anti-inflammatory properties. Interestingly, recent studies show that oxyresveratrol utilizes these properties to protect hair follicles from oxidative stress and promote hair regrowth [37].

Nevertheless, although the use of resveratrol and its derivatives as potential therapeutic agents has received considerable attention, the scientific literature suggests resveratrol action to be complex. Research suggests that frequent, small doses of resveratrol are more effective than a single large dose [38]. A major criticism is the low bioavailability of resveratrol, implying that high doses may be needed for therapeutic activity. At least one study, however, indicates that resveratrol could be regenerated through its metabolites [39].

Our goal is to correlate stilbene structural features to antioxidant experimental outcomes and to derive a chemical mechanism consistent with the stilbene superoxide scavenging activity. Earlier experimental work in our laboratory demonstrated that piceatannol is a better superoxide radical scavenger than resveratrol and the enhanced reactivity is attributed to its catechol moiety and the intermolecular hydrogen bond [40,41,42]. These findings were corroborated by standard electrochemical measurements of resveratrol and piceatannol scavenging of superoxide in the aprotic solvent, N,N-dimethylformamide, by Nakayama [43]. Theoretical studies also agreed with our results [44,45,46,47,48,49] in recognizing the resveratrol 4′-hydroxyl group as more reactive than those in positions 3 and 5. The dominant pathway for free radical scavenging by resveratrol derivatives is H-atom transfer over single-electron transfer [50,51].

In addition, we investigate the possible role of vitamin C (ascorbic acid) in these scavenging reactions. It is known that vitamin C functions chiefly as a single-hydrogen atom donor, while its radical anion, monodehydroascorbate, interacts predominantly with free radicals [52].

With this research study, using both experimental and computational methods, we extend our analysis to develop a DFT-derived superoxide scavenging reaction mechanism displayed by resveratrol, piceatannol and oxyresveratrol. The Solvation Model based on density (SMD) method was not included in our DFT calculations [53]. The originality of our approach is that we use hydrodynamic voltammetry to produce the superoxide radical *in situ* and then measure the antioxidant scavenging action directly. This electrochemical method underscores the fundamental chemistry and aids in elucidating a reaction mechanism by which polyphenolic natural compounds can scavenge free radicals. Although DPPH and ABTS assays are widely used for measuring antioxidant activity, they possess limitations including that they use non-biological nitrogen-centered radicals [54]. No prior work has explained the stilbene antioxidant mechanisms using DFT in correlation with rotating ring–disk electrode (RRDE) data. Through this procedure, we show how resveratrol, when acting as an antioxidant, could potentially regenerate itself through catalytic action and a renewed interpretation of resveratrol bioavailability may be necessary. Interestingly, the polyphenolic antioxidant mechanism shows a correspondence with that of the MnSOD enzyme [3].

## 2. Results and Discussion

Our laboratory has been deliberating a more in-depth analysis of the superoxide scavenging mechanism by polyphenols and considered that vitamin C could be a potential proton source for the superoxide dismutase scavenging mechanism [reaction (1)] in biological systems as it is an abundant species widely distributed in the body [55].

2O_2_^●−^ + 2H^+^ → O_2_ + H_2_O_2_ (1)

To obtain an enhanced perspective of the antioxidant mechanism, we considered also the action of an additional proton from a second molecule of vitamin C and a second molecule of superoxide. We suggest that this combination could make some scavengers act as mimics of superoxide dismutase, whose reaction is shown in (1) [56]. The metalloenzyme MnSOD removes (dismutates) two molecules of O_2_^●−^: first, a Mn^2+^ ion is used to reduce the first O_2_^●−^ to H_2_O_2_ and second, a Mn^3+^ ion oxidizes the second O_2_^●−^ to O_2_ [3]. In our case, with polyphenolic organic molecules, the first superoxide is reduced by capturing a hydroxyl H atom from the scavenger, while the action of the second superoxide differs markedly by undergoing oxidation through a π-π interaction [57]. Basically, the second superoxide radical releases its unpaired electron to the aromatic system of the scavenger and generates a molecule of O_2_.

An experimental study of the O_2_^●−^ interaction with resveratrol (Res) by N derivatives was performed by Murias and it was determined that the reaction occurs via H-atom transfer in two steps, reactions (2) and (3) [58].

Res + O_2_^●−^ → Res-4′ ^●^ + HO_2_^−^ (2)

HO_2_^−^ + H^+^ → H_2_O_2_ (3)

Previously, our computational studies demonstrated the importance of 4′OH to resveratrol scavenging [39] and this was later confirmed [59]. The formation of H_2_O_2_ arising from a reaction involving a molecule of superoxide, resveratrol H4′ atom, and an extra proton was described for resveratrol using ESR [58], confirmed with DFT including piceatannol [41] and supported by other studies [42,43].

Stacking π-π interactions are crucial in biological chemistry [60] and essential to our studies in explaining the polyphenolic scavenging mechanism. The literature shows examples of π-π interactions between resveratrol and/or its derivatives to biologically important molecules [61,62] including various amino acids [21,63,64]. Moreover, DNA base pair π-π stacking to stilbene derivatives is known [65]. Interestingly, researchers used DFT calculations and molecular dynamics to study how planar compounds like resveratrol stack with graphene and graphene oxide [66]. The study found that resveratrol formed the strongest π-π interactions with graphene, achieving a binding distance of 2.929 Å, significantly shorter than the normal 3.50 Å spacing found in graphite. These data support our DFT-derived scavenging mechanism.

### 2.1. RRDE Results

The rotating ring–disk electrode (RRDE) is a hydrodynamic electroanalytical method that was introduced by us [67] to quantify the antioxidant capability of molecules that scavenge the superoxide radical, which is generated *in situ*. It is a cyclovoltammetry variant, where besides the working electrode that performs reduction of bubbled O_2_ in the electrovoltaic cell (O_2_ + e^−^ → O_2_^●−^), there is a rotating electrode, provided by a positive fixed potential, which can reverse the same reaction.

#### 2.1.1. Resveratrol

**Figure 2:**
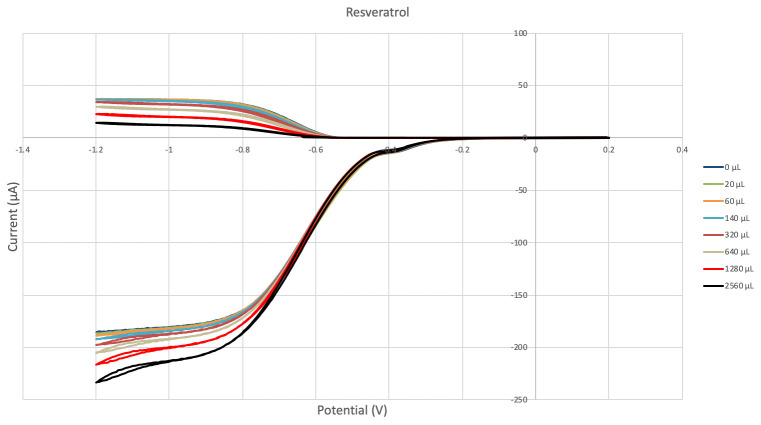
Voltammograms of 0.03 M resveratrol.

**Figure 3:**
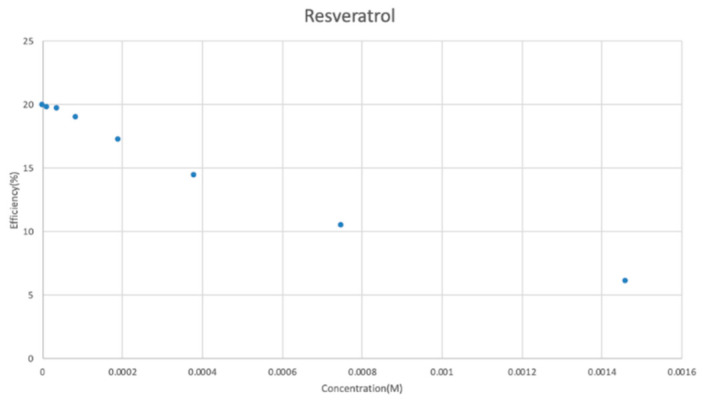
Collection efficiency of 0.03 M resveratrol.

**Figure 4:**
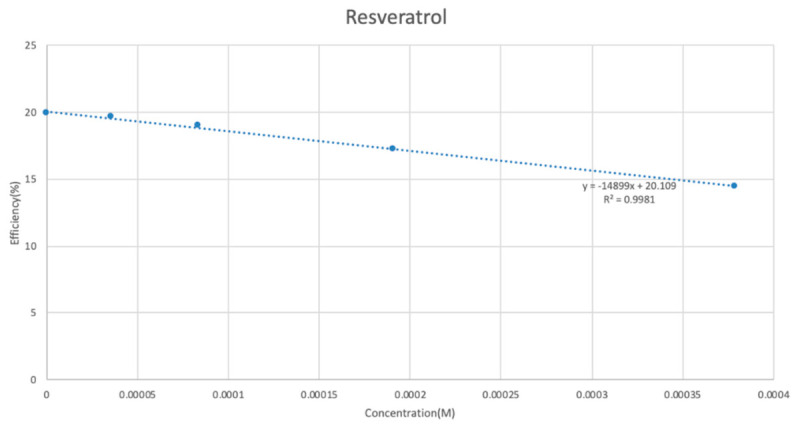
The first 5 spots of Figure 3 are selected as they express linear behavior. The slope of −1.5 × 10^4^ M^−1^ measures the antioxidant capability of 0.03 M resveratrol.

The stock 0.03 M resveratrol solution was used and Figure 2 shows the superimposed voltammograms of successive experiments obtained after taking a blank (black line) followed by tests where increasing aliquots of the antioxidant resveratrol were added to the electrovoltaic cell. Each color corresponds to a single experiment; for instance, the green lines correspond to the voltammogram obtained after adding the first aliquot (20 µL). The upper part of the figure reflects the amount of superoxide detected (at the rotating ring electrode). The value of the current for the green experiment is less than the blank, indicating that some superoxide has been eliminated by the 20 µL aliquot. As the subsequent second, third, etc., resveratrol volume aliquots are added, and antioxidant resveratrol concentration increases, the upper part of the signal (current) decreases, indicating lower superoxide concentration. An inverse relationship therefore exists, with the decrease in superoxide concentration correlating with increase in antioxidant concentration. Figure 3 shows the collection efficiency, which graphs the quotient between the current values for oxidation (upper part) and reduction (lower part) of Figure 2 versus total concentration of antioxidant. Thus, the first added aliquot of 20 µL (green) corresponds to the second point (immediately after the blank) in Figure 3, whereas the next point describes the efficiency of first plus second aliquots [(20 + 40) µL total antioxidant amount]. The equation of the line is −14,899 x + 20.109, R^2^ = 0.9981 and the slope of −1.5 × 10^4^ M^−1^ measures the antioxidant capability of 0.03 M resveratrol. We have observed that for some antioxidants, the efficiency curve, corresponding to Figure 3, is linear, whereas for other scavengers of superoxide there is asymptotic behavior. Regardless, the first few aliquots (about five) always show linear behavior, due to the low concentration of antioxidant, so that the scavenger reacts only with superoxide. The initial linear behavior is an excellent indication of antioxidant ability. For resveratrol the first five spots were selected, and the resulting line has a slope of −1.5 × 10^4^ M^−1^, shown in Figure 4. Indeed, our experiments show that decreasing values of superoxide concentration (corresponding to upper part of voltammogram) can be correlated to the amount of antioxidant added. The collection efficiency, obtained by using the initial low antioxidant concentration values, shows that the steeper the slope, the greater the rate of change, and the more effective the antioxidant. 

To evaluate how vitamin C affects resveratrol scavenging, we added three aliquots (20, 40, and 60 μL) of 0.03 M vitamin C to 120 μL of 0.03 M resveratrol solution and recorded the RRDE measurements and observed a much better efficiency. Combining vitamin C and resveratrol created a stronger antioxidant effect. The results are shown in Figures S1 and S2.

#### 2.1.2. Piceatannol

**Figure 5:**
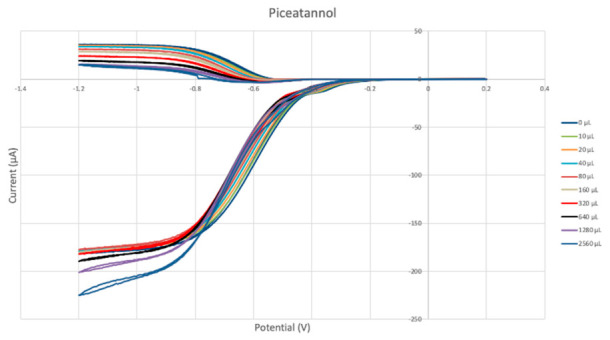
Voltammograms of 0.02 M piceatannol.

**Figure 6:**
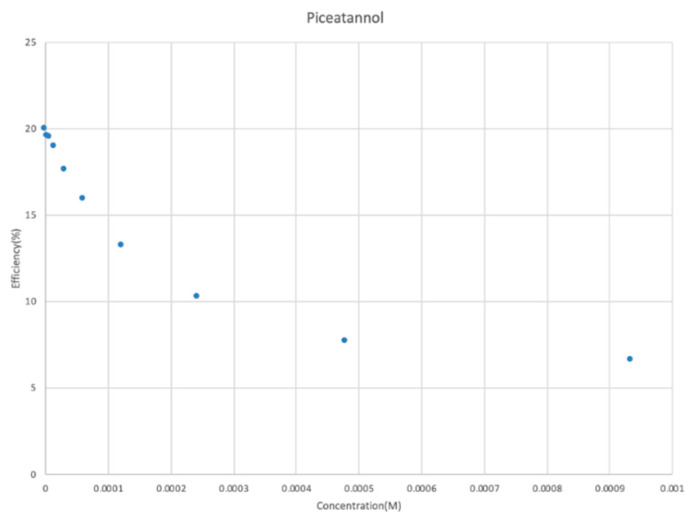
Collection efficiency of 0.02 M piceatannol.

Voltammograms (Figure 5) and collection efficiency (Figure 6) of 0.02 M piceatannol show an overall asymptotic non-linear behavior and so only the first six spots from Figure 6 were selected to express the linear behavior to calculate the collection efficiency, with equation of line as −66,070 x + 19.907, R^2^ = 0.9924, Figure S3. The slope of −6.6 × 10^4^ M^−1^ measures the antioxidant capability of piceatannol, which is about four times higher than that of resveratrol.

The effect of vitamin C addition on piceatannol scavenging was measured using RRDE by adding three aliquots (20, 40, and 60 μL) of 0.03 M vitamin C to 40 μL of 0.02 M piceatannol solution. The efficiency was calculated and, again, we see that combining vitamin C with piceatannol created a greater antioxidant effect. The results are shown in Figures S4 and S5.

#### 2.1.3. Oxyresveratrol

Voltammograms (Figure S6) and the collection efficiency plot (Figure S7) of 0.03 M oxyresveratrol provide the linear expression −13,728 x + 21.214, R^2^ = 0.9556. The slope of −1.4 × 10^4^ M^−1^ measures the antioxidant capability of oxyresveratrol, Figure S7**,** about the same as resveratrol. The effect of vitamin C addition on oxyresveratrol scavenging was measured using RRDE by adding three aliquots (20, 40, and 60 μL) of 0.03 M vitamin C to 40 μL of 0.03 M oxyresveratrol solution. Again, we see that vitamin C addition enhances the measured antioxidant effect. The results are shown in Figures S8 and S9.

### 2.2. DFT

#### 2.2.1. Piceatannol

As shown previously [41], the reduction of one superoxide anion through a scavenging of superoxide by piceatannol is more efficient than that of resveratrol because stabilization of the generated semiquinone 4′ piceatannol complex is helped by the intramolecular H-bond formed through H3′. It was also shown that formation of H_2_O_2_ was feasible when the semiquinone–HO_2_^−^ complex later interacted with a proton. In this work, we explore if the same reaction could be performed by another proton source: vitamin C, a vital and important organic acid in biological systems.

**Figure 7:**
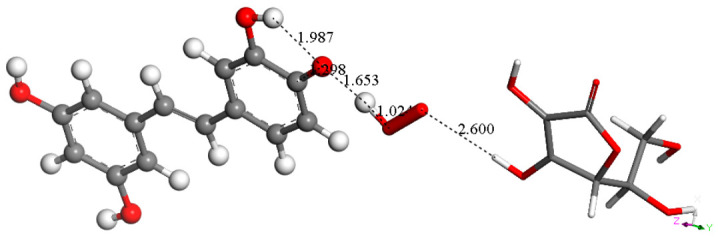
After geometry optimization of O(superoxide), poised at van der Waals separation (2.60 Å) from piceatannol H4′, the latter is abstracted, O4′---H = 1.653 Å, and captured by superoxide, O-H bond length = 1.024 Å. From this arrangement, vitamin C, right, is placed at van der Waals distance (2.60 Å).

**Figure 8:**
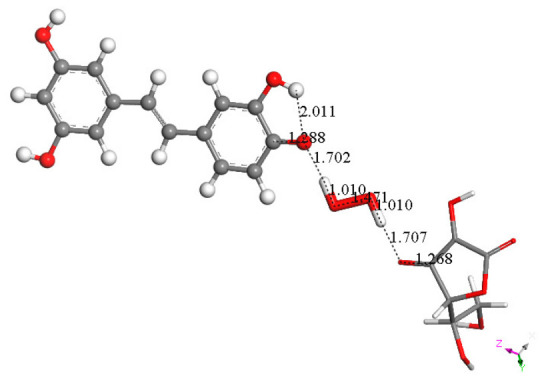
Geometry optimization of Figure 7 arrangement shows formation of H_2_O_2_, well separated from piceatannol–semiquinone radical anion, 1.702 Å, and ascorbate, 1.707 Å. ΔG for reaction involving Figure 7 and Figure 8 is −34.7 kcal/mol.

**Figure 9:**
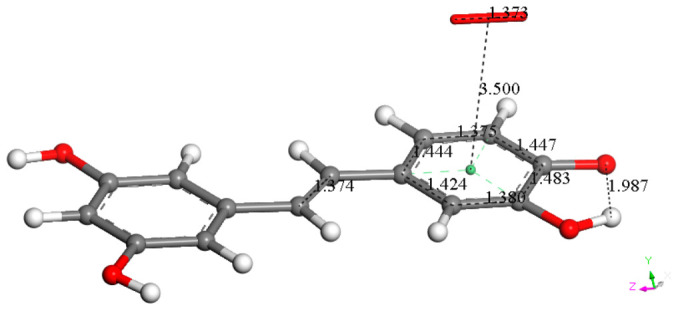
After elimination of H_2_O_2_ and ascorbate from Figure 8, marked differences are observed in the aromatic C-C bond distances, due to double-bond delocalization (range: 1.375–1.483 Å), as C2′-C3′ (1.380 Å) and C5′-C6′ (1.375 Å) are shorter than C1′-C2′ (1.424 Å), C3′-C4′ (1.483 Å), C4′-C5′ (1.447 Å) and C1′-C6′ (1.444 Å). A second superoxide (O-O bond length of 1.373 Å) is placed π-π at van der Waals distance, 3.50 Å, from the semiquinone ring.

**Figure 10:**
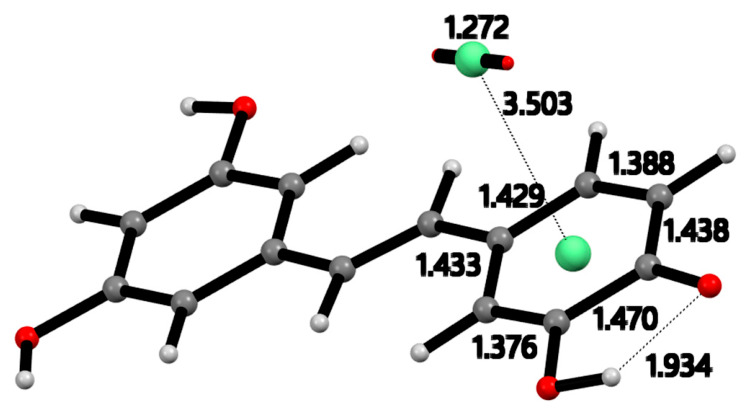
DFT minimization of Figure 9 (a non-radical anionic complex) results in the dioxygen species being formed (O-O bond distance of 1.272 Å, consistent with a molecule of O_2_ and shorter than the superoxide distance of 1.373 Å seen in Figure 9) and removed from polyphenol interaction, 3.503 Å. ΔG for reaction involving Figure 9 and Figure 10 (electron transfer) is −14.6 kcal/mol.

**Figure 11:**
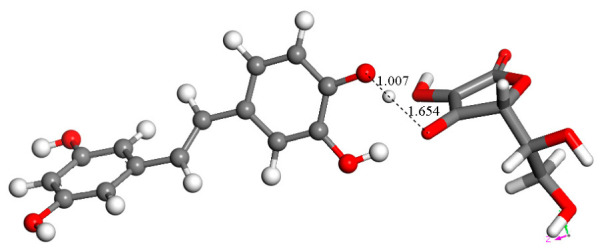
After elimination of O_2_ from Figure 10, a vitamin C molecule is poised at 2.60 Å from semiquinone O4′ anion and, after geometry optimization, piceatannol is reestablished due to capture of the acidic vitamin C proton, O4′-H = 1.007 Å. Ascorbate is also produced in this final calculation. The overall reaction is that shown in Equation (4). ΔG for reaction involving the proton transfer is −13.8 kcal/mol.

**Figure 12:**
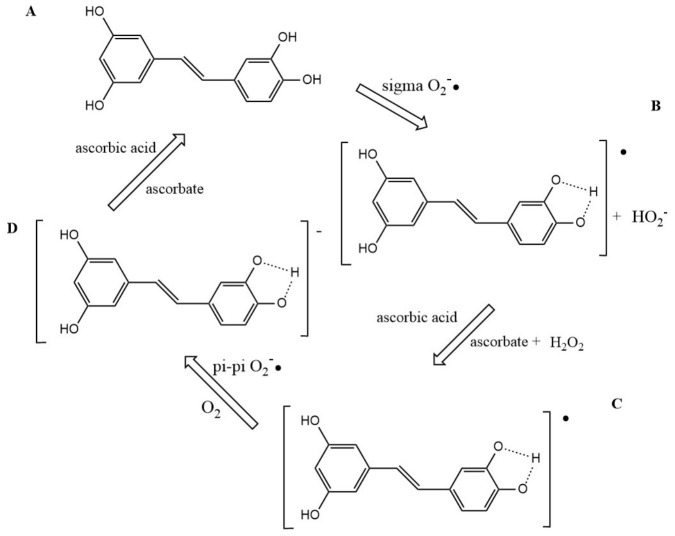
Piceatannol is regenerated after the following steps: (**A**): sigma superoxide interacts with H4′ forming HO_2_^−^ anion plus the semiquinone piceatannol radical (Figure 7); (**B**): the semiquinone radical reacts with vitamin C to give H_2_O_2_ plus ascorbate (Figure 8); (**C**): the semiquinone radical reacts in π-π manner to give a semiquinone anion plus a molecule of O_2_ (Figure 10); (**D**): vitamin C gives its proton to the semiquinone anion to restore piceatannol (Figure 11).

After extracting the H4′ proton from piceatannol, the HO_2_^−^ anion is poised at van der Waals separation 2.60 Å [1.40 Å (O) + 1.20 Å (H)] from vitamin C (Figure 7 shows the initial position), and a geometry optimization shows formation of H_2_O_2_, ascorbate and piceatannol–semiquinone anion radical, Figure 8. The first two species were eliminated and the remaining semiquinone radical was π-π posed for reaction with a second superoxide radical. The van der Waals association between these two radicals, Figure 9, showed an easy reaction after geometry optimization that was caused by an electron transfer from the superoxide to the ring and which liberated a molecule of O_2_, Figure 10. Thus, the unpaired electron of superoxide entered the aromatic system, very probably helped by the extended stilbene planarity. The other product of this reaction is an anionic species that interacts with a second molecule of vitamin C, Figure 11, and prepares piceatannol for additional scavenging. The entire piceatannol mechanistic scheme is shown in Figure 12. This overall reaction (4) is closely related to that performed by the SOD metalloenzymes (1).

2 O_2_• ^−^ + 2 C_6_H_8_O_6_ (ascorbic acid) → O_2_ + H_2_O_2_ + 2 C_6_H_7_O_6_^−^ (ascorbate) (4)

#### 2.2.2. Oxyresveratrol

The resveratrol derivative oxyresveratrol, Figure 1, found in mulberries, has an additional hydroxyl in position 2′ of ring B, instead of position 3′ in piceatannol. We are interested in observing the effect of this chemical variation on the antioxidant scavenging of the superoxide radical and focus our attention on the oxyresveratrol 4′OH first and then the 2′OH.

##### Oxyresveratrol 4′Hydroxyl

**Figure 13:**
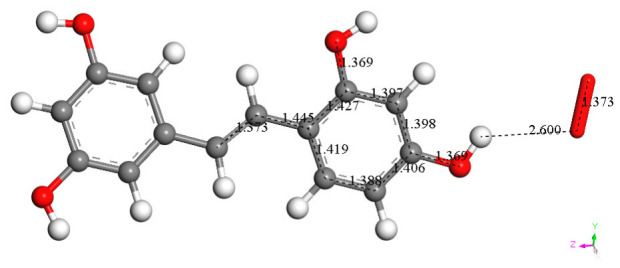
Superoxide approaches H4′ hydroxyl of oxyresveratrol, with van der Waals separation of 2.60 Å. This is an anionic (−1) radical arrangement.

**Figure 14:**
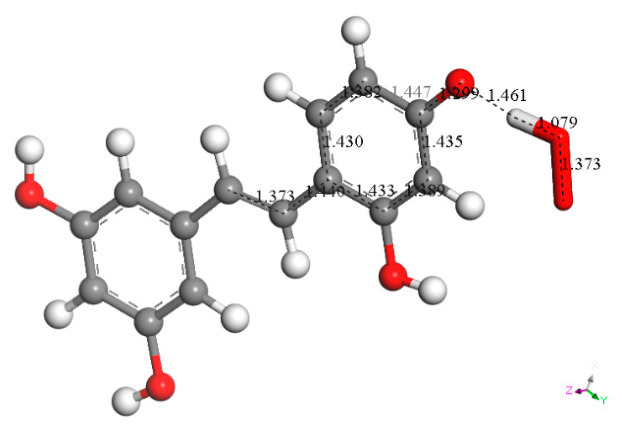
Geometry optimization of Figure 13 radical anion arrangement shows capture of H4′ by O(superoxide). The formed HO_2_^−^ anion species is well separated from the semiquinone oxyresveratrol radical, 1.461 Å. ΔG for reaction involving Figure 13 and Figure 14 is −64.6 kcal/mol.

**Figure 15:**
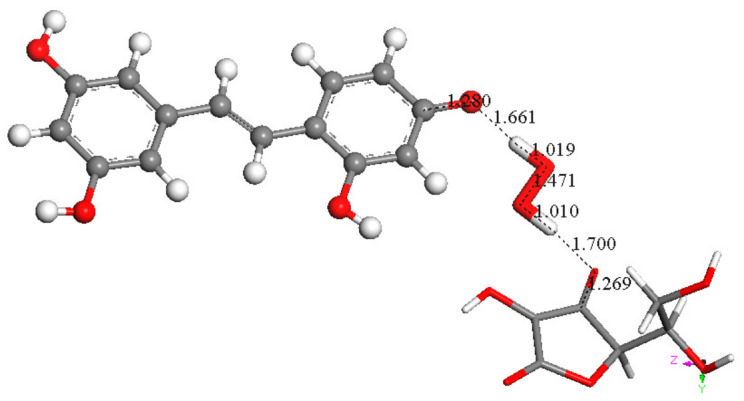
Geometry optimization of Figure S10 arrangement shows formation of H_2_O_2_, well separated from ascorbate, 1.700 Å, and 4′-oxyresveratrol semiquinone radical, 1.661 Å. ΔG for reaction involving the proton transfer is −61.8 kcal/mol.

**Figure 16:**
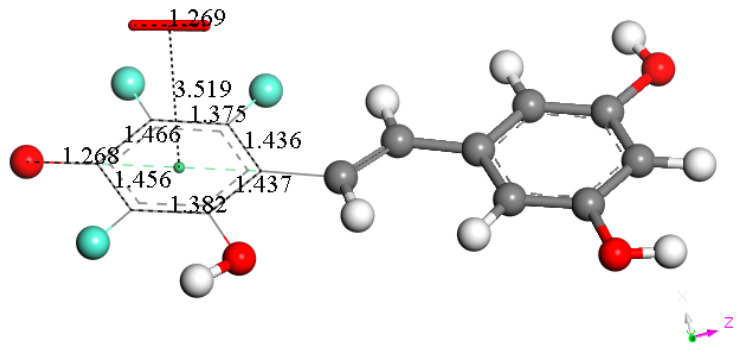
Outcome after elimination of H_2_O_2_ and ascorbate from Figure 15 assemblage, and π-π placement of superoxide with the aromatic ring. The resulting DFT optimization shows formation of O_2_ and a semiquinone anion whose features on the B ring are determined by the presence of the short C4′-O4′ bond, 1.269 Å. ΔG for reaction involving this electron transfer is −36.7 kcal/mol.

A molecule of superoxide was poised at van der Waals separation, 2.60 Å, from H4′, Figure 13. The corresponding geometry optimization shows capture of H4′ by O(superoxide) forming a HO_2_^−^ anion plus oxyresveratrol semiquinone radical, separated by 1.461 Å, Figure 14. Similar to piceatannol, we proceeded with the approach of vitamin C, Figure S10, that can release its proton to the HO_2_^−^ anion to form H_2_O_2_, Figure 15. After elimination of H_2_O_2_ and ascorbate from the Figure 15 assemblage, superoxide was placed in a π-π van der Waals (3.50 Å) arrangement with the aromatic ring. Its geometry optimization shows the species on top of the ring far removed, e.g., separation of 3.519 Å (slightly longer than van der Waals separation) between ring centroid and superoxide centroid, Figure 16. More importantly, the bond length of the original superoxide radical, 1.371 Å, becomes shorter, 1.269 Å, and is ascribed to formation of a molecule of O_2_. Thus, the unpaired electron of superoxide has entered the aromatic system, very probably helped by the extended stilbene planarity. This is confirmed by the aromatic C-C bonds (range 1.378–1.466 Å), greater than the one shown in Figure 13 (1.397–1.427 Å). In addition, two very short double bonds C2′-C3′ (1.378 Å) and C5′-C6′ (1.382 Å) are conjugated with the short carbonyl C4-O4′ (1.268 Å) bond length. Also, the range of long C-C bonds is markedly different, 1.436–1.466 Å. Thus, the semiquinone features of the B ring are determined by the presence of the short C4′-O4′ bond. This figure shows a non-radical anionic configuration.

**Figure 17:**
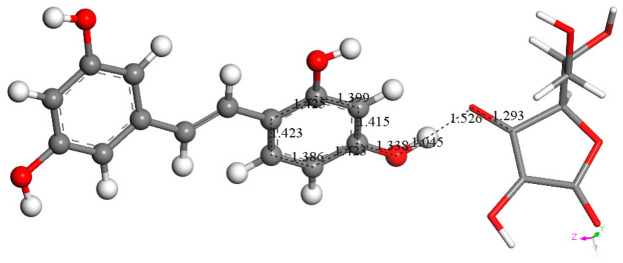
Result of geometry optimization after elimination of O_2_ from Figure 16 arrangement, and introduction of a second vitamin C molecule placed at van der Waals distance from O4′ of the remaining semiquinone radical, 2.60 Å. Here, we see oxyresveratrol is regenerated, and the ascorbate anion is separated at 1.526 Å and having a carbonyl C-O double-bond length of 1.293 Å. ΔG for reaction involving this proton transfer is −64.6 kcal/mol.

After elimination of O_2_ from Figure 16, a DFT geometry optimization was performed. Next, the second vitamin C was introduced, and an additional DFT geometry optimization was completed which reconstituted oxyresveratrol, Figure 17. This is similar to what occurred with piceatannol. The whole mechanism regarding the 4′OH of oxyresveratrol, reaction (4), is equivalent to that shown by SOD, (1).

##### Oxyresveratrol, 2′OH Moiety

Calculations involving 2′-hydroxyl of oxyresveratrol were attempted using the same pathway as for 4′OH oxyresveratrol and piceatannol. Initial calculation of superoxide showed no hydrogen atom capture of H2′. Superoxide is not able to abstract H2′ from oxyresveratrol (ΔG = 2 kcal/mol) and so vitamin C is placed pointing its proton to the O(superoxide) not involved in the H-bond to H2′, Figure S11. The approach of vitamin C was able to donate a proton to the superoxide in the superoxide–oxyresveratrol complex of Figure S12. However, there was no effective H_2_O_2_ formation, indicating that H2′ was not abstracted from oxyresveratrol, Figure S12. Indeed, theoretical results show a more acidic environment makes H_2_O_2_ formation feasible, Figure S13, when reacting an H^+^ (proton) in place of vitamin C (in Figure S12). We conclude that vitamin C is too weak an acid to help with removal of oxyresveratrol-H2′.

#### 2.2.3. Resveratrol

**Figure 18:**
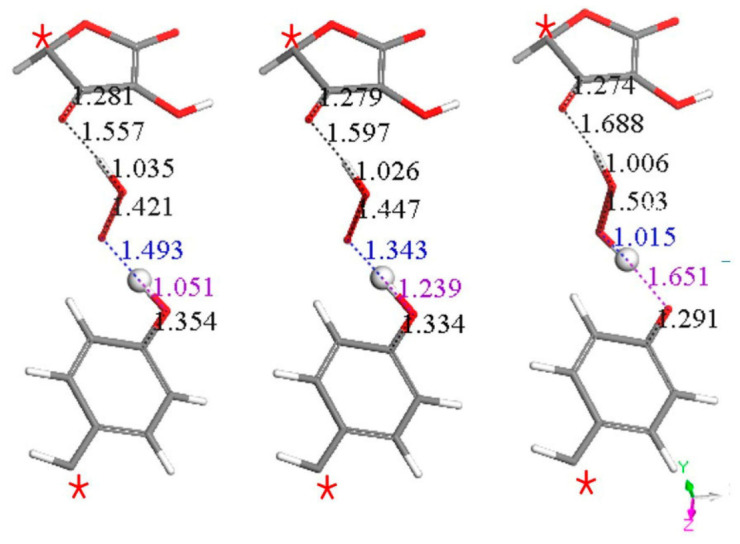
The molecular environment of the proton transfer; stars denote points of truncation (vitamin C, top, and resveratrol, bottom). The reactants (**left**), TS (**center**) and products (**right**) of vitamin C approaching the resveratrol–superoxide complex. Asterisks mark the positions where the molecule extends to form the complete structures shown in Figure S14.

**Figure 19:**
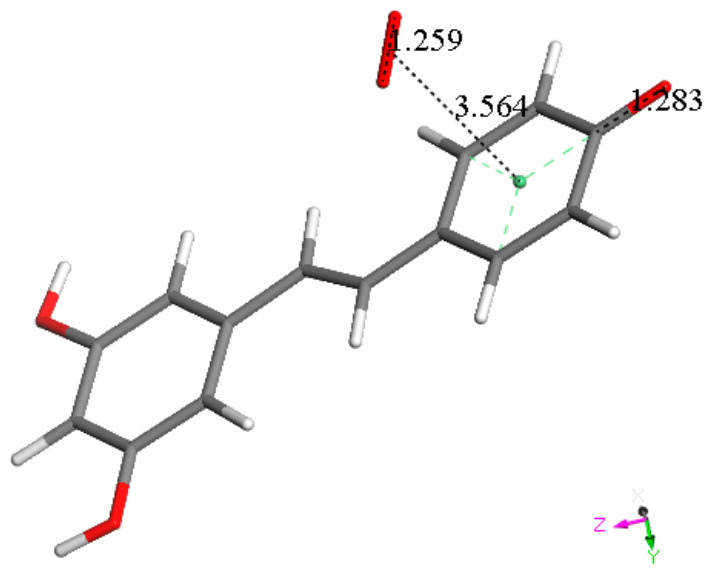
The optimized result of Figure S16 arrangement shows a molecule of O_2_ (O-O distance of 1.259 Å) separated from ring B, 3.564 Å. The C4′-O4′ distance, 1.283 Å, is closely related to the equivalent distance in Figure 18 right, 1.291 Å. ΔG for reaction involving electron transfer is −30.4 kcal/mol.

**Figure 20:**
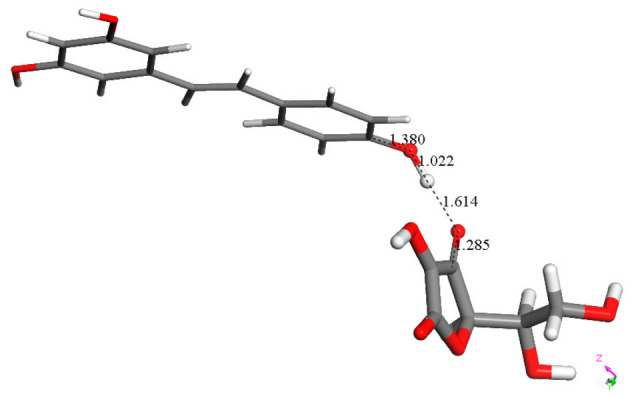
Final calculation for resveratrol after introducing a second molecule of vitamin C into the arrangement (O_2_ excluded) of Figure 19. This shows resveratrol reformation plus a well-separated ascorbate, 1.614 Å. ΔG for reaction involving the proton transfer is −42.7 kcal/mol.

Previous studies [40,41] showed that a proton can react with the semiquinone resveratrol–superoxide complex to form a distinct H_2_O_2_ species; we now explore if vitamin C can perform the same reaction. We positioned the labile proton of vitamin C near the most exposed oxygen atom of the resveratrol–superoxide complex, 2.60 Å, and performed a geometry optimization. The results, shown in Figure 18, identify that a transition state (TS) is needed, ΔG = −5.1 kcal/mol and E(barrier) = 2.4 kcal/mol. Details of TS are shown in Figures S14 and S15. Consequently, the distinct H_2_O_2_ and ascorbate species of Figure 18 (right) were eliminated for further geometrical minimization, and the remaining radical was paired with a second superoxide anion (O-O distance of 1.373 Å) that was placed at 3.50 Å van der Waals separation in a π-π manner, Figure S16. The outcome, shown in Figure 19, produced a non-radical anionic resveratrol derivative due to the transfer of the superoxide π electron to the resveratrol B ring. The resultant molecule of O_2_ was eliminated and the remaining anionic species was approached by a second vitamin C molecule, with its labile proton directed towards O4′. Final geometry optimization (Figure 20) confirms the reformation of resveratrol and a distinct ascorbate, 1.614 Å, the whole process follows reaction (4), equivalent to (1).

## 3. Materials and Methods

### 3.1. Materials for RRDE Solutions

Tetrabutylammonium bromide, dried TBAB (Sigma-Aldrich, St. Louis, MO, USA); dimethyl sulfoxide, DMSO, anhydrous, ≥99.9% (Sigma-Aldrich).

Resveratrol (Indofine Chemical Co., Hillsborough, NJ, USA), C_14_H_12_O_3_. RRDE solution: concentration of 0.03 M (0.064 g in 10 mL DMSO), clear, reddish yellow.

Piceatannol (Indofine Chemical Co.), C_14_H_12_O_4_. RRDE solutions: concentrations of 0.02 M (0.046 g in 10 mL DMSO), clear, yellow.

Oxyresveratrol (Indofine Chemical Co.), C_14_H_12_O_4_. RRDE solution: concentration of 0.03 M (0.069 g in 10 mL DMSO), clear, yellow.

### 3.2. Hydrodynamic Voltammetry (RRDE)

The experiments were performed using the Pine Research rotating ring–disk electrode (RRDE) and the WaveDriver 20 bipotentiostat (Pine Research, Durham, NC, USA) with the Pine Research MSR Electrode Rotator, which controls the rotation speed to ensure well-defined mass transport via convection. The ring–disk electrode is composed of two electrically isolated electrode surfaces. The electrode tip was an E6R1 ChangeDisk with a fixed gold ring and gold disk insert (Pine Research). To remove potential film formation before its use, the ring/disk Au/Au working electrode tip was polished using a 0.05 µm alumina suspension (Pine Research). In RRDE, the bipotentiostat measurements are obtained at the same time using both the currents at the disk and ring electrodes (that correspond to charge movements among the ring, the disk, and the counter-electrode) and the potentials of the disk and ring electrodes in relation to the single reference electrode [68]. Overall, the 5-neck electrochemical RRDE cell that was used contained four electrodes, the two gold rotating ring and disk working electrodes (Pine Research), one coiled Pt wire counter-electrode, and one Pt wire reference electrode. The electrochemical cell contained a 0.1 M (1.61 g) solution of dried TBAB dissolved in 50 mL of anhydrous dimethyl sulfoxide. The solution in the electrochemical cell was bubbled using a Pasteur pipette with dry O_2_/N_2_ (35%/65%) for five minutes, to establish the required dissolved molecular oxygen level. The partial oxygen-mixture gas tank was used to allow oxygen to flow into the voltaic cell at a comparable rate to that at which the antioxidant was scavenging the generated superoxide radical.

As a control, we performed cyclic voltammetry on 1 mM of each of the three stilbenes and 100 mM TBAB in DMSO at a glassy carbon electrode. Solutions were purged with N_2_ to remove oxygen prior to measurement and the scan rate was 100 mV/s. The results showing no stilbene electrochemical activity are in Figure S17.

The Aftermath software release 1.6.10523 (Pine Research Instrumentation, Durham, NC, USA) [69] was used to set up the parameters needed for the experiment: the potential sweep was applied to the disk from 0.2 V to −1.2 V and then reversed to 0.2 V, while the potential of the ring electrode was held constant at 0.0 V. The disk potential was set to sweep at set intervals in the negative direction in order to reduce the superoxide radicals that reacted at the ring to oxygen molecules. The disk’s voltage sweep rate was set to 25 mV/s. The rotation setting used for the rotation of the Au/Au ring–disk electrode was chosen to be 1000 rpm at the disk electrode. The superoxide radical was generated through a molecular oxygen reduction, and the peak was detected at around −0.6 V. Meanwhile, the reverse-oxidation reaction of the remaining unreacted superoxide radicals was detected at the ring electrode.

An initial “blank” solution consisting of bubbled O_2_, the electrolyte TBMB, and DMSO was run, and the voltammogram showing the current vs. potential graphs was recorded using the Aftermath software (Pine Research). Next, an initial antioxidant aliquot was introduced. The solution in the voltaic cell was bubbled with the gas mixture for 5 min, a new voltammogram was recorded, and the corresponding efficiency was calculated. In this manner, the rate at which the increasing concentration of the antioxidant scavenged the generated superoxide radicals during the electrochemical reaction was determined upon the addition of each antioxidant aliquot. The ratio of the ring–disk current was defined as “efficiency”. These data were later evaluated using Microsoft Excel and MATLAB R2025a [70]. The related RRDE graph indicates the volume amount used in each of the aliquots. Finally, a quantitative measure of the antioxidant activity of the samples is seen in the decreasing slope of the curve upon the incremental addition of the antioxidant and which describes the overall decrease in efficiency. Any decrease in the collection efficiency was expected to be due to the amount of superoxide consumed through the increasing antioxidant concentration [67].

### 3.3. Computational Study

DFT calculations were run using BIOVIA Materials Studio DMoL^3^, implemented in Materials Studio version 7.0 (Dassault Systèmes, San Diego, CA, USA). The results of these calculations make clear how a molecule’s 3D structure influences its antioxidant properties and scavenging behavior. The double numerical polarized (DNP) basis set, which includes all occupied atomic orbitals plus a second set of valence atomic orbitals, was used, providing double the numerical representation of the valence shell [71]. This basis set is analogous to a “double-zeta” Gaussian basis set (such as 6-31G) but it uses numerical atomic orbitals instead of analytical Gaussian functions. The basis set is augmented with polarized d-valence orbitals [72] and is used with all electrons treated explicitly (no pseudo-potentials), thus providing high accuracy for chemical bonding and molecular geometry. The BLYP functional is a generalized gradient approximation (GGA) that includes the Becke exchange with the LYP correlation functional [73].

We are looking mainly for trends in the barriers to identify fast and slow steps for correlation with our experimental studies, and these can be achieved using the simpler and faster BLYP functional. In addition, Grimme’s correction was applied when Van der Waals interactions were involved [74]. The solvent effect was determined in these calculations by using the continuous model of Dmol^3^ [75]. We compared the results from calculations done in DMSO and in water, to better mimic the RRDE environment, and did not see any variations. The real space cutoff of 5 Å was set for the numerical integration of the Hamiltonian matrix elements. The self-consistent field convergence criterion was established at less than 10^−6^ electron/Å^3^ for the root mean square variation in the electronic density. The convergence criteria employed during geometry optimization were 2.72 × 10^−4^ eV for energy and 0.054 eV/Å for force.

## 4. Conclusions

By examining resveratrol, piceatannol, and oxyresveratrol, this investigation, using experimental RRDE measurements and DFT calculations, clarifies the relationship between stilbene structure and an energy-feasible antioxidant mechanism. Consistent with our previous findings [41], RRDE measurements confirm that the catechol-containing piceatannol is four times more effective an antioxidant than resveratrol. Furthermore, the nearly identical RRDE efficiency values obtained for resveratrol and oxyresveratrol suggest that the additional 2′OH group in the latter is of little importance to its antioxidant capacity. These results coincide with our DFT analysis, which indicates that the 2′OH group does not significantly influence superoxide scavenging. This extra hydroxyl group may serve to enhance the compound’s aqueous solubility, however. Vitamin C addition to each of the three stilbenes studied enhanced superoxide scavenging through a mechanism consistent with that shown by superoxide dismutase (SOD) enzymes, yielding hydrogen peroxide H_2_O_2_ and molecular oxygen O_2_. That these common natural products obtained through diet may be able to function as SOD mimics to reduce free radical concentration in the body is a notable finding. While vitamin C serves as a proton donor to facilitate this process, it is insufficient to abstract the 2′OH group of oxyresveratrol, which requires a stronger acid source. Interestingly, our findings obtained from energetically feasible DFT calculations reveal that resveratrol acts catalytically, regenerating itself after free radical scavenging. This catalytic cycle implies that its low bioavailability may not hinder its effectiveness, since high concentrations are not needed for antioxidant activity. As we noted in the Introduction, research suggests that frequent, small doses of resveratrol are more effective than a single large dose [38]. This dosing pattern can be considered as consistent with moderate wine consumption, which may explain why the Mediterranean diet, characterized by regular wine intake and corroborated by measurements of phenolic urinary metabolites, is associated with lower CVD risk [29,30]. By using fundamental chemical and electrochemical behavior, this research bridges the gap between our understanding of how dietary compounds support redox balance in the body, fundamental to many disease states and aging. We hope our results can stimulate further biological/biochemical interest.

## Data Availability

The original contributions presented in this study are included in the article/supplementary material. Further inquiries can be directed to the corresponding authors.

## Supplementary Materials

The following supporting information can be downloaded at https://www.mdpi.com/article/10.3390/ijms27135691/s1.
