# Supplementary material for: Electrochemical and Computational Studies Show That Vitamin C Assists Resveratrol, Piceatannol and Oxyresveratrol in Superoxide Scavenging, Suggesting a Superoxide Dismutase Mechanism
Source: Int J Mol Sci. 2026 Jun 24;27(13):5691. doi: 10.3390/ijms27135691 (PMC13361674; doi:10.3390/ijms27135691)
Supplement: Supplementary file 1 [file ijms-27-05691-s001.zip › ijms-4304054-supplementary/ijms-4304054-supplementary.pdf]

Supplementary material deposited for

Electrochemical and Computational Studies Show That Vitamin C Assists Resveratrol, Piceatannol and Oxyresveratrol in Superoxide Scavenging Suggesting a Superoxide Dismutase Mechanism

Authors: Francesco Caruso, Taylor S. Teitsworth, Raiyan Sakib, Alessio Caruso, Stuart Belli, Miriam Rossi

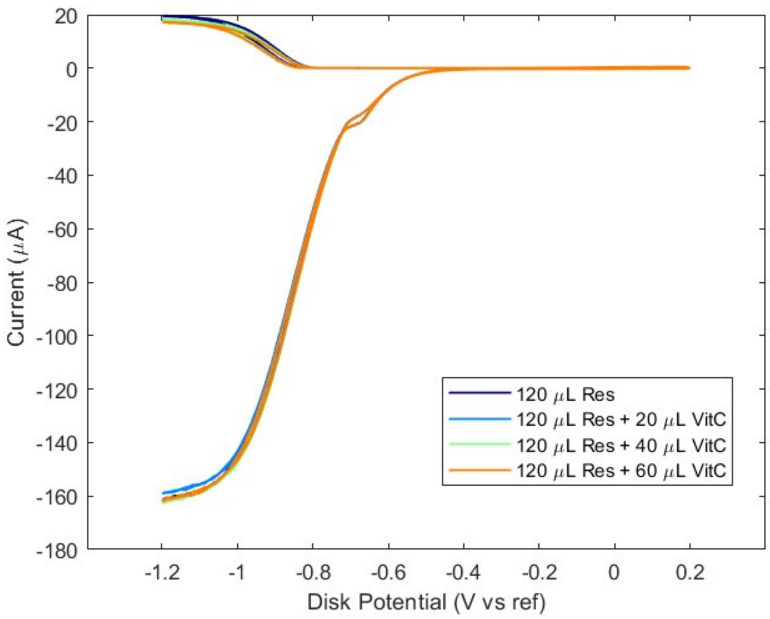

Figure S1. Voltammograms of an initial aliquot of resveratrol and later added vitamin C.

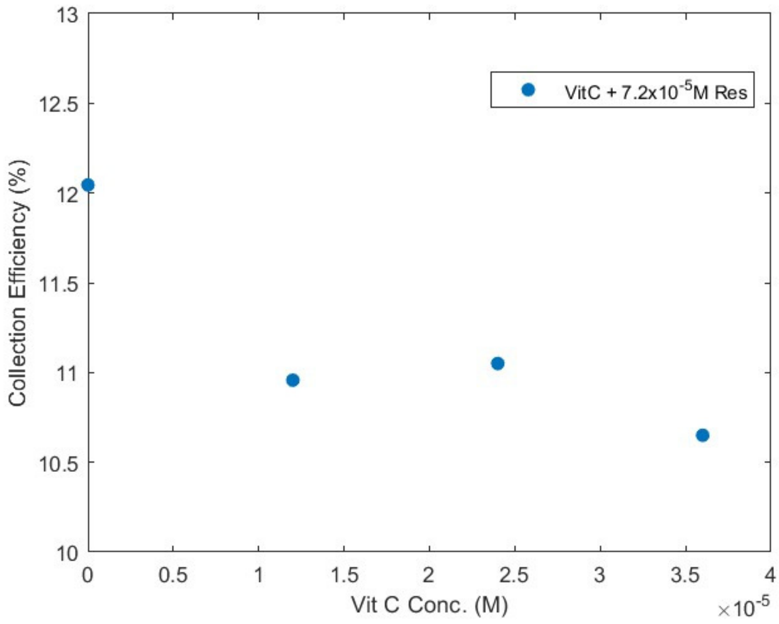

Figure S2. Vitamin C added to resveratrol shows a better collection efficiency (data obtained from Figure S1).

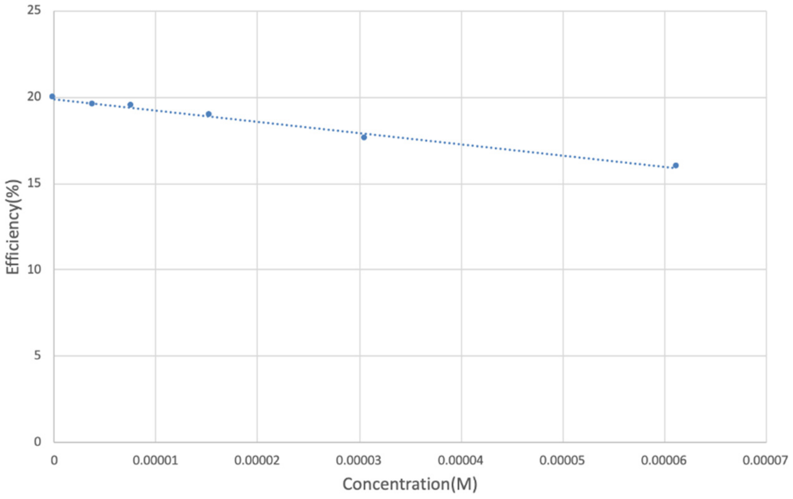

Figure S3. The first 6 spots of piceatannol **Figure 6** are selected to express the linear behavior needed for the calculation of collection efficiency, -66070 x + 19.907, $R^2 = 0.9924$. The slope of $-6.6 \times 10^4\ M^{-1}$ measures the antioxidant capability of piceatannol.

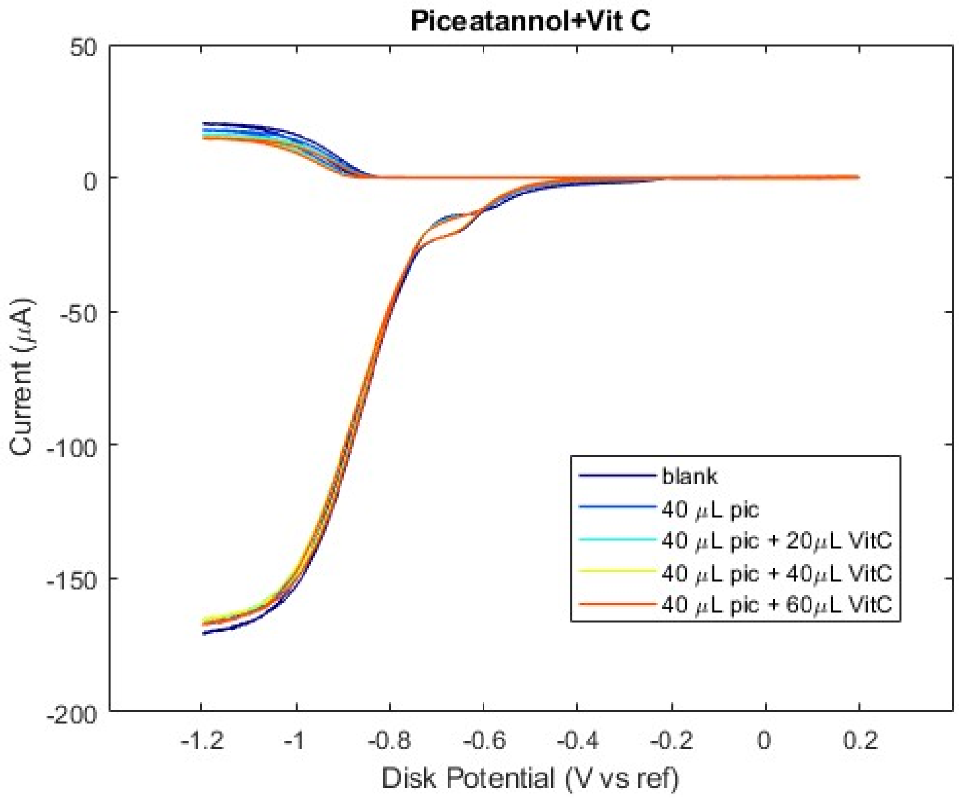

Figure S4. Voltammograms of an initial aliquot of piceatannol and later added aliquots of vitamin C.

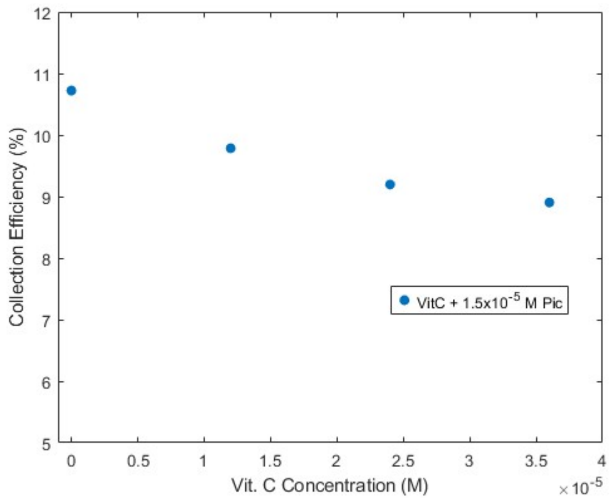

Figure S5. Vitamin C added to piceatannol shows a better collection efficiency (data obtained from Figure S4).

.

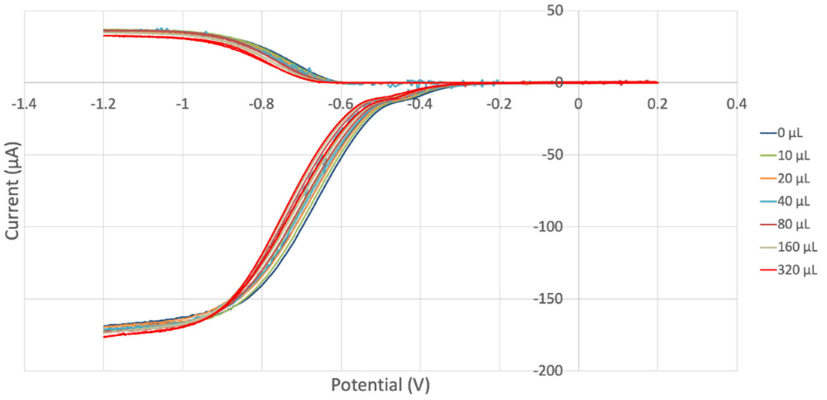

Figure S6. Voltammograms of 0.03 M oxyresveratrol.

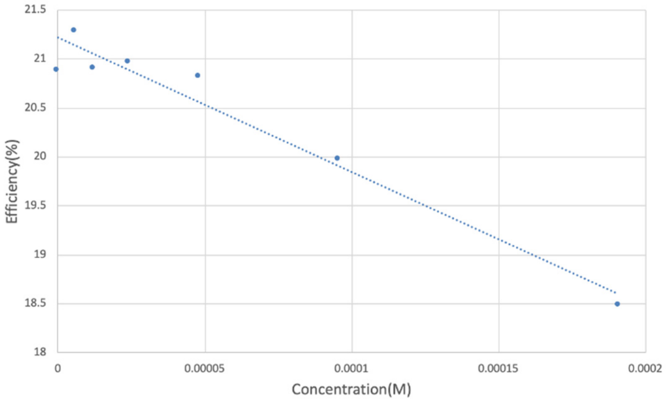

Figure S7. Collection efficiency of 0.03 M oxyresveratrol whose plot provides the linear expression -13728 x + 21.214, R^2 = 0.9556. The slope of -1.4 x 10^4 M^{-1} measures the antioxidant capability of oxyresveratrol.

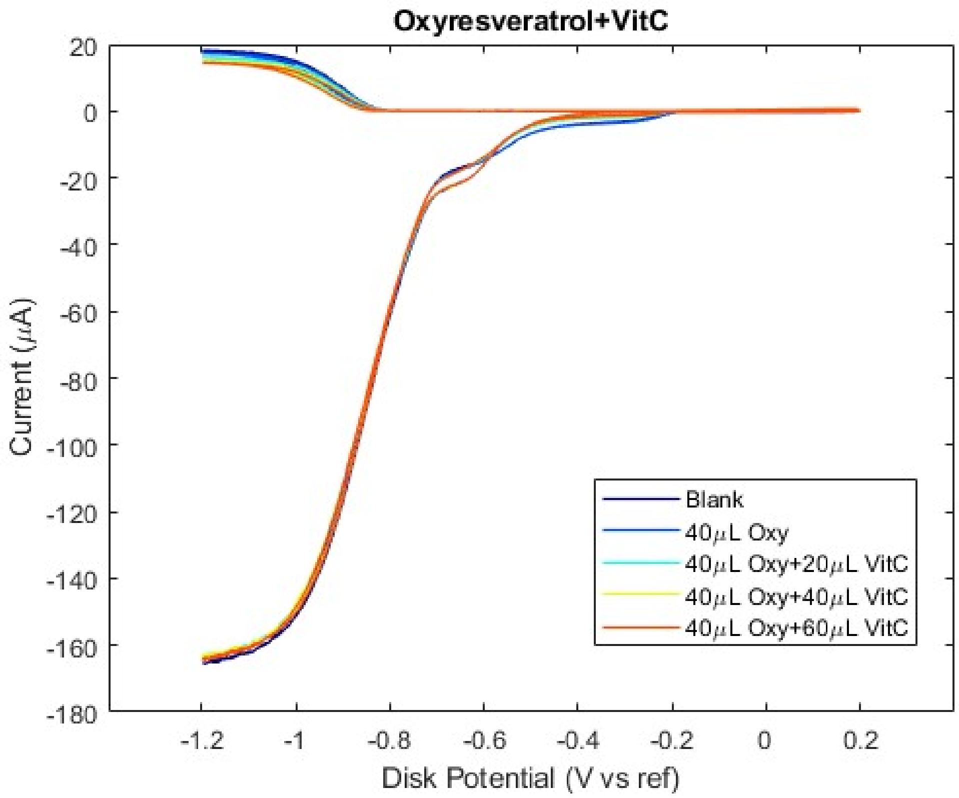

Figure S8. Voltammograms of an initial aliquot of oxyresveratrol and later added aliquots of vitamin C.

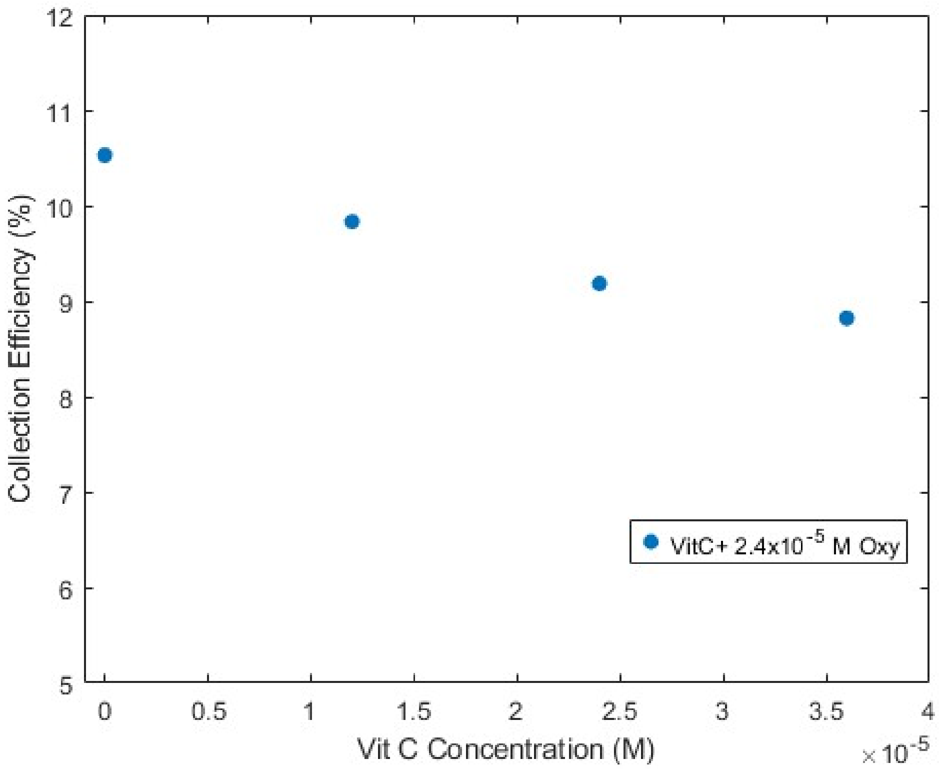

Figure S9. Vitamin C added to oxyresveratrol shows a better collection efficiency (data obtained from Figure S8).

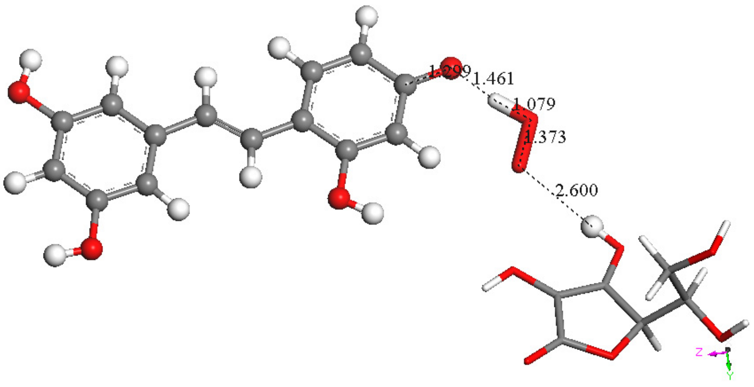

Figure S10. Ascorbic acid approaching the oxyresveratrol more exposed 4'O atom of HO_2^-, 2.60 Å, from Figure 14 configuration.

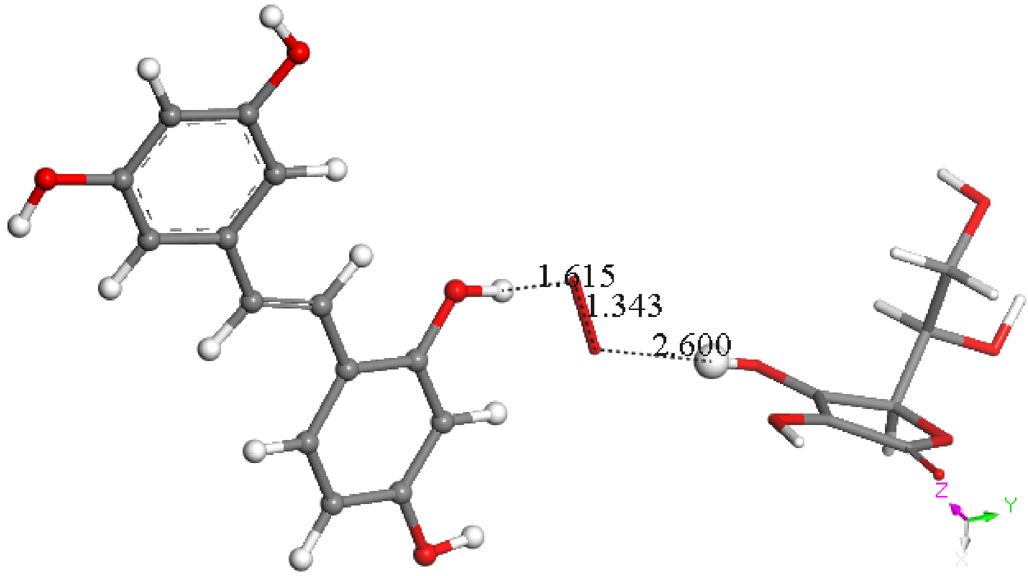

Figure S11. The initial state (oxyresveratrol) for a geometry minimization regarding H2' scavenging and the result is shown in Figure S12, below.

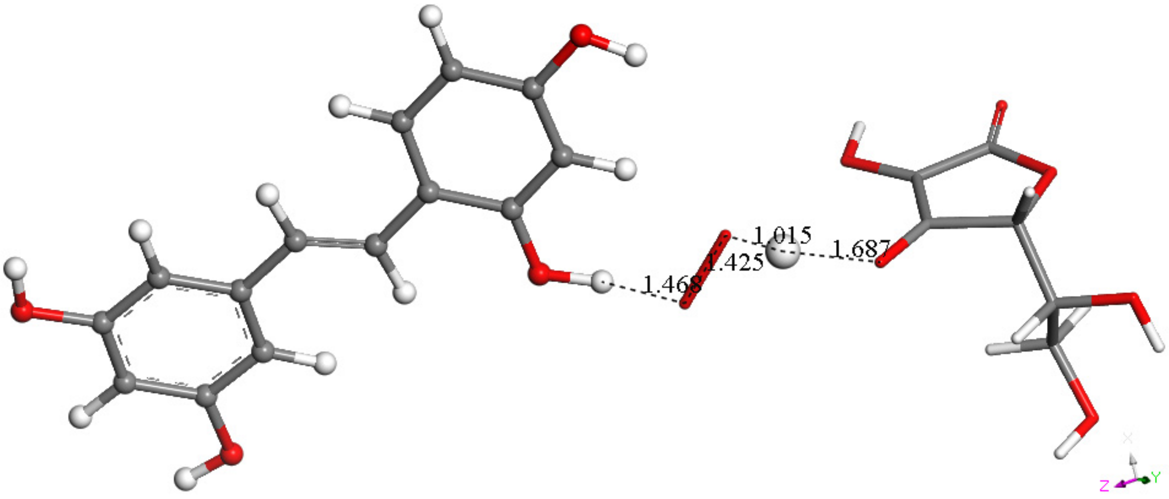

Figure S12. Result of geometry optimization from Figure S11 arrangement shows a different outcome, than shown in Figures 8 and 11: Ascorbic acid cannot abstract H2' from oxyresveratrol to form H_2O_2.

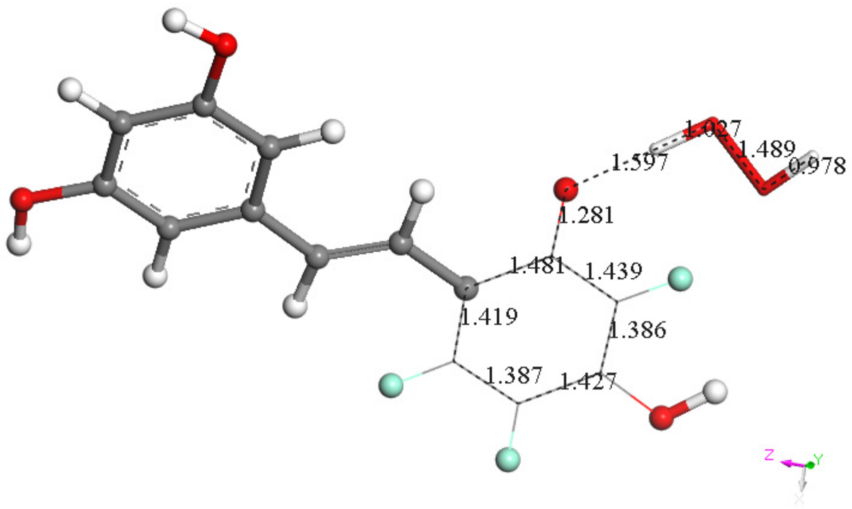

Figure S13. Result (oxyresveratrol) after using a proton instead of ascorbic acid, in the Figure S11 configuration, shows H_2O_2 formation, well separated from O2', 1.597 Å.

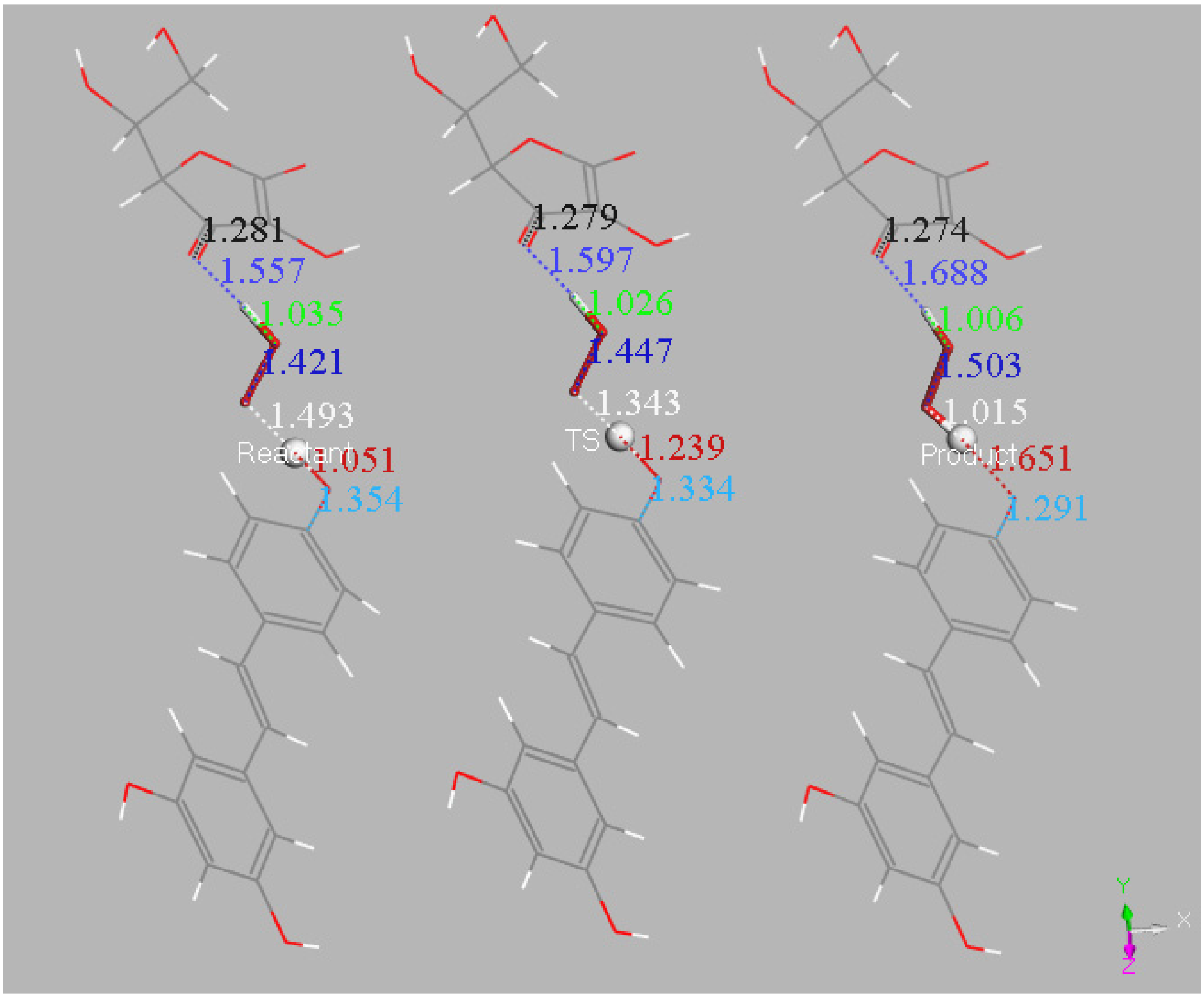

Figure S14. The molecular environment (complete structures) in the proton transfer for resveratrol scavenging of superoxide. The figure shows Reactants (left), TS (center) and Products (right) of vitamin C approaching the resveratrol-superoxide complex. (Details are in Figure 18.)

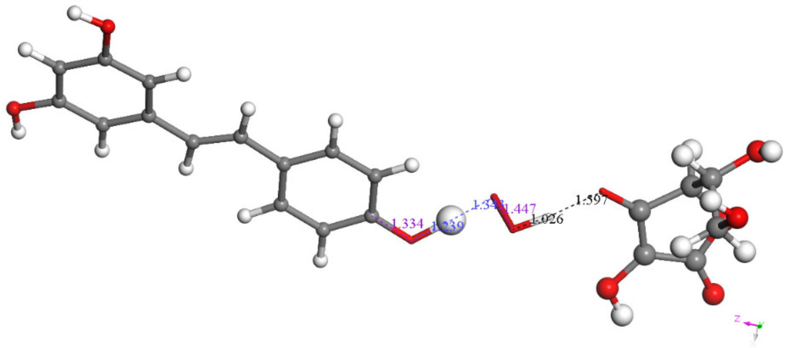

Figure S15. Details of TS for vitamin C approaching the resveratrol-superoxide complex.

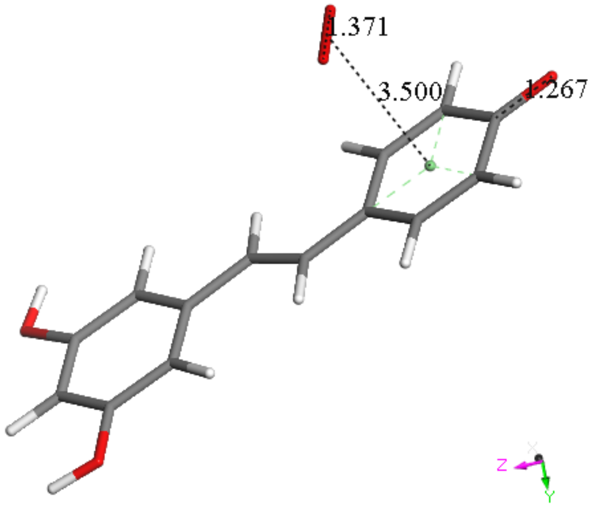

Figure S16. After ascorbate and H_2O_2 elimination from Figure 18 (right) a superoxide radical is poised by the B ring (resveratrol).

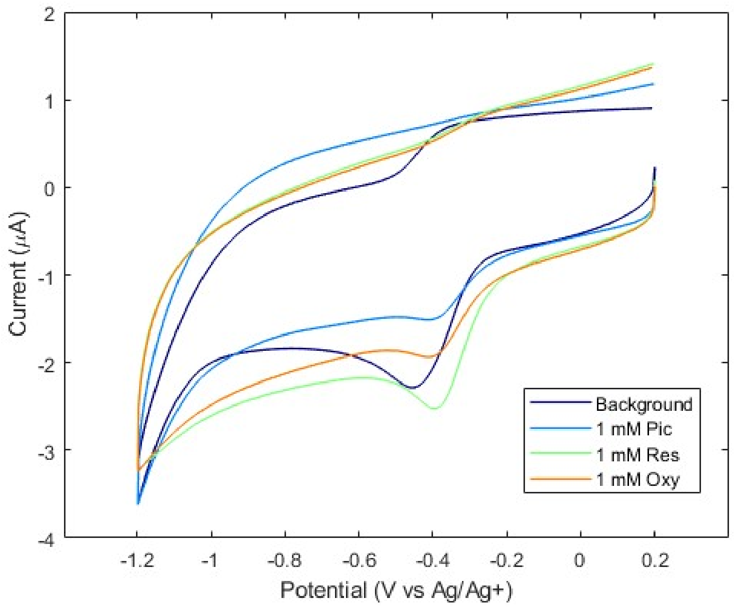

Figure S17. Cyclic voltammetry of 1 mM stilbene and 100 mM TBAB in DMSO at a glassy carbon electrode. Solutions were purged with N_2 to remove oxygen prior to measurement, and the scan rate was 100 mV/s
